# Income and Sex Moderate the Association Between Population Density and Reproduction: A Multilevel Analysis of Life History Strategies Across 23 Nations

**DOI:** 10.1007/s10508-024-02955-w

**Published:** 2024-07-22

**Authors:** Jose C. Yong, Chun Hui Lim, Peter K. Jonason, Andrew G. Thomas

**Affiliations:** 1 Present Address: Department of Psychology, Northumbria University, Northumberland Building, Northumberland Road, Newcastle Upon Tyne, NE1 8ST UK; 2 Department of Psychology, Stanford University, Stanford, CA USA; 3 Psychology Research Institute, University of Economics and Human Sciences, Warsaw, Poland; 4 School of Psychology, Swansea University, Swansea, UK

**Keywords:** Reproduction, Fertility, Life history strategy, Population density, Resource competition, Sex differences

## Abstract

While previous studies guided by evolutionary life history theory have revealed several important socioecological moderators of the influence of population density (PD) on reproduction, absent is an understanding of how individual-level factors such as personal resources and sex differences might interact and play a role. Using data from a large sample of clients (*N* = 4,432,440) of an online dating company spanning 317 states nested within 23 countries, we contributed a robust multilevel analysis of life history effects by assessing the interaction between state-level PD and individual-level income on offspring quantity, and we further qualified this analysis by sex. Consistent with previous research, PD was negatively correlated with having children. Consistent with our novel hypotheses, this negative relationship was moderated by income such that the link between PD and low fertility became weaker with increasing levels of income and these patterns were stronger for men than for women. These results held despite controlling for a variety of country-level, state-level, and individual-level confounds. Findings are discussed together with theoretical and practical implications for the management of fertility based on evolutionary life history perspectives.

**Supplementary Information:**

The online version contains supplementary material available at 10.1007/s10508-024-02955-w.

## Introduction

Overcrowding and reproduction have become increasingly urgent topics given the doubling of the world’s population over the last half century and fertility rate problems that threaten the sustainability of nations (Bergaglio, 2017). On the one hand, developing nations are experiencing problems with overpopulation and scarcity of resources (Cassils, 2003); on the other hand, industrialized nations are experiencing birthrate declines that create problems associated with aging populations and declining productivity (Jarzebski et al., 2021). These various issues highlight the need for a better understanding of the factors that underlie global reproductive rates.

One factor that has garnered increasing research attention is population density (PD), with multiple human and non-human studies showing an inverse link between PD and fertility (e.g., de la Croix & Gobbi, 2017; French et al., 1965; Loftin & Ward, 1983; Lutz et al., 2006; Wright et al., 2019). A framework that has been used to understand this link is the resource-competition view of life history theory (Rotella et al., 2021; Sng et al., 2017). From this perspective, high PD intensifies competition for scarce resources and opportunities, thus prompting individuals to respond by “slowing down” and becoming future-oriented, such as investing in education, engaging in committed long-term mating (as opposed to unrestricted short-term mating), marrying later, and having less children but investing more in them to enhance their later-life competitiveness (Sng et al., 2017; Yong et al., 2024).

Nevertheless, findings on the dynamics of PD and fertility have been mixed. For instance, a study on 174 countries confirmed the PD-fertility link but found that this link was attenuated by socioecological factors such as environmental harshness (Rotella et al., 2021), while another study of 122 countries failed to find a relationship between PD and adolescent fertility (Luoto, 2019a). Other studies also suggest that the links between high PD, slow strategy, and low fertility are not so straightforward. Studies that examined the moderating effects of childhood circumstances indicate that people’s life history strategy can be calibrated by their developmental experiences to be faster or slower in a trait-like manner, which in turn functions to adaptively guide behavioral responses to life stressors or affordances (Griskevicius et al., 2013; Rickard et al., 2014; Tan et al., 2022). According to this line of work, slow strategists respond to stressors (e.g., mortality cues, economic uncertainty) by being more cautious and taking fewer risks, whereas fast strategists respond by being more impulsive and taking more risks, reflecting distinct strategies to either delay gratification or immediately exploit opportunities to cope with challenges. Such findings suggest that whether an environmental factor like PD slows reproduction depends on individual-level factors which influence how organisms respond to environmental threats and opportunities.

To address these gaps and build on previous work that sought to identify additional variables in the PD-fertility dynamic (e.g., Figueredo et al., 2021; Kuzawa & Bragg, 2012; Luoto, 2019a, 2019b; Luoto et al., 2019; Minkov & Bond, 2015; Rotella et al., 2021), we conducted a novel test of the availability of resources as an individual-level moderator of the link between state-level PD and individual-level fertility. As competition for limited resources has been proposed to explain people’s inclinations toward long-term orientation and slower reproduction (Sng et al., 2017), we suggest that income, which indexes whether people possess adequate personal resources, may moderate the adoption of a slower strategy when confronted with high PD. In addition, we extended this analysis to account for the evolutionarily guided view that financial resources have a greater impact on men’s (versus women’s) mating and reproductive success (Buss, 1989; Fieder & Huber, 2022; Li et al. 2013; Yong et al., 2022) by testing for sex differences in our hypothesized relationships.

To facilitate a well-powered test of the interactions between PD, income, and sex on fertility, we acquired data from a leading online dating company with services around the world and covariate data from secondary sources. The final sample comprised approximately 4.4 million subjects from 317 states within 23 countries, which provided data on (1) individual-level income, sex, and number of children, (2) state-level PD, and (3) individual-, state-, and country-level control variables. Through this investigation, we provide a robust validation of the relationship between PD and fertility while contributing a novel examination of the moderating effects of income and sex.

### Life History Strategies

Life history theory broadly argues that organisms’ limited budget of energy and time imposes on them a trade-off between somatic effort (i.e., growth and maintenance) and reproductive effort (i.e., mate seeking and reproduction; Ellis et al., 2009; MacArthur & Wilson, 1967; Stearns, 1992). Although each of these efforts carries fitness benefits when pursued successfully, they often come at the expense of one another—for instance, time spent pursuing mates cannot be used to search for food or care for offspring. As such, organisms—including humans—must prioritize their energetic investments according to some adaptively preferable pace of reproduction. This prioritization, termed life history strategy (LHS), can be conceptualized as a trade-off between faster versus slower reproduction (Sæther, 1987) and has implications for how soon an individual sexually matures and has their first offspring, as well as how many offspring they will have and the quality of parental investment each offspring will receive (Del Giudice et al., 2015; Ellis et al., 2009; Figueredo et al., 2006). These LHSs appear to be heritable to some extent (Flatt & Heyland, 2011; Figueredo et al., 2004, 2020). Data from the national LHS genetic factor index, which includes genes associated with temporal orientation, risk tolerance, and other LHS outcomes (e.g., AR, DRD4, 5-HTTLPR VNTR; see Minkov & Bond, 2015), showed that countries with higher rates of adolescent fertility have a faster LHS genetic factor index while countries with more complex economies (indicative of stability and long-term planning) have a slower LHS genetic factor index (Luoto, 2019b).

Variations in ecological factors, which imply the need for different optimal energy allocation strategies, play a significant role in selecting for particular LHS phenotypes and calibrating the reproductive pace of organisms (Kozlowski & Weigert, 1986; Tooby & Cosmides, 1992). A classic set of ecological factors that has been argued to drive LHS variations is the stability and harshness of the environment (Ellis et al., 2009; Neuberg & Sng, 2013), whereby “hopeful” (i.e., secure and predictable) environments incentivize long-term planning and investment in the self and offspring because such environments afford resident organisms greater control over their own mortality, whereas “desperation” (i.e., harsh and unpredictable) environments render investments in an uncertain future unsensible; instead, reproducing as soon or as much as possible while focusing less on offspring quality will be more profitable (Daan & Tinbergen, 1997).

Humans have been documented to follow this variation in LHS. Although humans, when compared with other species, generally adopt a slow LHS characterized by long developmental periods, heavy investment in a few offspring, and long lifespans (Kaplan et al., 2000), some within-species variation exists. For example, studies of neighborhoods in Chicago (Wilson & Daly, 1997) and 373 counties in the USA (Griskevicius et al., 2011a) showed that higher levels of harshness based on lower life expectancy and violent crime were associated with faster LHS, including earlier onset of reproduction and having more children. In contrast, delayed or slower reproduction in safer and more predictable environments was found to be linked with markers of increased parental investment, such as reduced child mortality, increased literacy of parents and children, higher socioeconomic status, and preferences for fewer children (Bongaarts, 2002; Ellis, 2004; Engelhardt & Prskawetz, 2004; Westoff, 1992).

More recent investigations have nevertheless raised the issue of whether “harshness” is too broad or simplistic as a descriptor of environments. For instance, one study found that climatic harshness measured via ambient cold is associated with lower fertility (Luoto, 2019a), thus suggesting that different types of environmental harshness may impact LHS in distinct ways (André & Rousset, 2020). Research has also indicated that the effects of harshness on LHS may go in opposing directions at individual versus population levels (see Pollet et al., 2014). For example, studies using individual-level harshness (e.g., childhood abuse, dysfunctional family units) often show that harshness predicts faster LHS (e.g., Mell et al., 2018), but when population-level harshness (e.g., extrinsic mortality) is used, harshness was found to predict faster or slower LHS depending on other factors such as childhood socioeconomic status (Griskevicius et al., 2011a, 2011b; Rickard et al., 2014) and density-dependent competition (e.g., André & Rousset, 2020), thus indicating that the connection between harsh environments and fast LHS is not as straightforward at the population level as research suggests at the individual level. Taken together, these findings highlight the role of ecological factors in shaping reproductive pace and, thus, trade-offs between mating and other motives, but more research is also warranted given the mixed findings on how the environment may interact with individual-level factors to influence reproductive variance.

### Population Density, Competition, and Fertility

Another ecological factor that has been identified as having an influence on fertility is PD. The impact of PD on fertility was first reported in non-human studies where higher PDs were found to predict lower reproductive rates in various domestic and wild animals (Christian, 1961, 1971; Fowler, 1981, 1987; French et al., 1965; Wright et al., 2019). Experimental studies further confirmed a causal relationship between increasing PD and the downregulation of reproduction across a range of species (Both, 1998; Dhondt et al., 1992; Leips et al., 2009). Researchers have drawn from these insights to understand human reproductive variance and similarly found that fertility decreases as a function of increasing PD in human samples (Allen et al., 2008; Firebaugh, 1982; Loftin & Ward, 1983; Lutz et al., 2006; Sinervo et al., 2000; but see Luoto, 2019a for exceptions).

A resource-competition view of LHS has been used to explain how PD affects fertility (Sng et al., 2017, 2018). This approach stresses that when PD is low and there is little competition for resources, organisms will enact a fast, quantity-driven strategy (e.g., having more offspring sooner) to quickly exploit available resources. By contrast, in densely populated environments where inhabitants must fight for resources which are necessarily limited, those lacking the ability to compete will be unable to acquire crucial resources needed for survival and reproduction. Hence, organisms in such environments are hypothesized to adopt a slow strategy, delay reproduction, and focus on long-term investment in the accumulation of competitive capacities (e.g., building competencies and achieving social status) to improve their likelihood of success at competing for resources and opportunities.

Data from several investigations support these hypothesized patterns. Sng et al. (2017) examined the relationship between PD and LHS by comparing between countries (Study 1) and between states in the USA (Study 2) and found that as PD increased, people were more likely to plan for the future (e.g., greater proportion of people investing in retirement), orient toward committed long-term relationships, have children later, have fewer children, and invest in children’s development (e.g., higher rates of preschool enrolment). These results were independent of potential confounds like economic development, urbanization, and population size. The researchers then experimentally tested the underlying role of future orientation: in Study 3, participants either read an article that described populations as increasing in density or read nothing, while in Study 4, participants listened to either crowd conversation noise or white noise, after which their preferences for a smaller reward sooner or a larger reward later were recorded. Results showed that participants exposed to stimuli indicating dense and crowded populations were indeed more likely to prefer delayed but larger rewards relative to participants who were not exposed to any such stimuli, thus supporting future orientation as the mechanism by which PD slows reproduction and restricts fertility.^1^

While Sng et al.’s (2017) research confirmed the links between PD and slower LHS and addressed the hypothesized role of future orientation, they did not examine one important element of the theory—whether these patterns of delayed reproduction and increased investment in the self and offspring emerge from competition for scarce resources. Other studies help to fill this gap. For example, people who live in societies that place a premium on prestigious or well-paying jobs but perceive stiff competition for such jobs have been documented to hold less favorable attitudes toward marriage and prefer having less children (Yong et al., 2019, 2024). Another study revealed that people with strong materialistic motives (i.e., valuing, striving, and competing to acquire status-denoting material possessions such as luxury goods) viewed marriage and having children more negatively than did people who were less materialistic (Li et al., 2011). These various findings suggest that in highly competitive environments like modern and economically advanced cities, endeavors such as furthering education, earning money, and gaining and demonstrating status are prioritized over reproductive goals, causing people to devalue and put off dating, marrying, and having children. When slow strategists do have children, they feel compelled to invest substantial effort in parenting to ensure that their children can meet the competitive demands of society (Ellis et al., 2009; Yong & Li, 2021).

### Moderating Factors

While the literature reviewed thus far (e.g., Lutz et al., 2006; Sng et al., 2017) has elucidated the links between PD, LHS, and reproductive rate, other studies that have failed to find these links (Luoto, 2019a) or highlighted the factors that moderate them paint a more complex picture. For example, Rotella et al. (2021) found that the relationship between PD and fertility weakened as living conditions became harsher (e.g., higher rates of homicide and pathogens). The authors theorized that PD-induced competition might take on more lethal forms under such conditions, which would in turn amplify the harshness of those conditions and shift preferences toward faster LHS such as having more children and investing less per child. People’s childhood developmental experiences have also been observed to calibrate their LHS such that they persist into adulthood and prompt distinct responses to similar environmental cues (Rickard et al., 2014). When participants were exposed to stressors such as economic uncertainty, resource scarcity, or increased mortality, those who grew up in safer and more stable childhood environments responded by slowing down and being more cautious, whereas those with harsher and less stable childhood experiences discounted the future, acted more impulsively, and engaged in riskier behaviors (e.g., Griskevicius et al., 2011b, 2013; Mittal & Griskevicius, 2014; Tan et al., 2022). This suggests that how people respond to environmental affordances depends on their individual capacities, such as whether they possess the means to overcome difficulties or exploit opportunities. Correspondingly, the impact of PD on people’s LHS, in particular their proclivities toward faster or slower reproduction, may be moderated by factors that influence their ability or need to compete. We considered the role of two such possible moderators—whether people have sufficient personal resources as well as sex differences in the importance of resources for mating effort.

#### Availability of Resources

As PD has been theorized to reduce fertility because competition for scarce resources spurs resident organisms to delay reproduction while focusing on developing and maintaining competitiveness (Sng et al., 2017, 2018), whether people have adequate resources can influence their vulnerability to the fertility-slowing effects of PD. More specifically, individuals who lack resources may feel more compelled to prioritize building their competitive capacities (e.g., furthering education, pursuing a career, gaining social status) over other pursuits (e.g., mate seeking, starting a family) in order to contest more effectively for resources compared to individuals who already have them (Yong et al., 2024). This obsessive need for resources is not trivial because individuals with resources have more means to pursue their reproductive interests than individuals without resources. For example, marriage is a strong predictor of having children and wealthier people (in particular the men) are more likely to be married (Aloni, 2018; Fieder & Huber, 2022). Furthermore, given the large investments in offspring that are needed within competitive, high-PD environments (Sng et al., 2017), the rich are less constrained in the number of children they can viably raise (Bar et al., 2018). These dynamics lead to differential fertility between the haves and have-nots: although preoccupations with competition for resources can come at the expense of fertility, individuals who have successfully acquired resources—via competition or otherwise (e.g., inheritance)—remain more likely to have children (Yong et al., 2024) and are in a better position to have more if they wish (Bar et al., 2018; Nitsche et al., 2018). Therefore, although the greater somatic investments required by PD-induced competition reduce fertility at the aggregate level of society, investments in competition still increase fertility at the level of individuals who win and possess the resources needed for mating and reproduction.

Taken together, the availability of resources is expected to moderate the negative relationship between PD and fertility. Individuals who lack resources will be more reproductively hindered because producing and nurturing offspring is a resource-heavy endeavor. In turn, those who perceive themselves as not having enough resources—particularly in high-PD environments—may adaptively focus on resource competition while putting off mating and having children, leading to an increasingly steeper negative association between PD and fertility as a function of decreasing personal resources.

#### Sex Differences

Research has shown that the impact of resource competition on reproductive outcomes is stronger for men than for women (Yong et al., 2019). According to evolutionary theories of mating, men are especially concerned about their social status and resourcefulness because of women’s preferences for these aspects in romantic partners (e.g., Buss, 1989; Li, 2007). These concerns and preferences are not unfounded as studies have found that wages positively predict the likelihood of being married for men but not for women (with a stronger effect of ever being married on reproduction in men than in women; Fieder & Huber, 2022), higher socioeconomic status (e.g., college education, employment, homeownership) is associated with a transition to parenthood and more offspring for men but not for women (Lim, 2021), and women who were married to men of lower income faced a pronounced increase in childlessness (Huber et al., 2010). As men face greater pressure than women on being able to compete for and acquire sufficient wealth and resources, we predicted that the relationship between high PD and reduced fertility will be stronger for men. In addition, as having resources has a greater bearing on men’s mating success, the moderating effect of resource availability on PD and fertility is also expected to be stronger for men.

### The Current Research

Based on the foregoing analysis, the current study hypothesized a three-way interaction effect of availability of resources, PD, and sex on fertility. As income is a common modern proxy for resources, we used self-reported income to operationalize people’s perceptions of the availability of resources. Arguably, having more income would signal that one has more resources, which would then reduce the need to invest in competition for resources and allow other objectives like mating and reproduction to be pursued. Thus, we predicted that income would moderate the well-established inverse relationship between PD and fertility, such that the negative correlation between PD and offspring quantity will be stronger when income is lower. In addition, we predicted a moderating effect of sex such that these patterns of results will be more pronounced for men than for women.

To test these predictions, we used data from a large sample of clients of an international, online dating company operating in 23 countries to facilitate a multilevel analysis of the interaction effect of income (individual-level factor) and PD (state-level factor) on number of children (individual-level outcome), thus affording sufficient variability in PDs and enabling a well-powered study. In addition, we controlled for an array of potential confounds including country-level gross domestic product (GDP) and economic inequality (Gini), state-level GDP, and individual-level demographics of age, sex, and education.

## Method

### Subjects

Data for this study were provided by Spark Networks Services GmbH (formerly Affinitas), which operates in more than 20 countries under different names (e.g., EliteSingles, eDarling). Members of the dating sites run by the company are predominantly heterosexual (96.0%) single adults seeking a long-term, committed relationship. The company provided data for each country through Excel files, with the largest samples (e.g., USA, Germany, France) containing membership records for over 1 million individuals. In total, the initial sample exceeded 9.5 million.

The data were cleaned to remove subjects with missing data on our key variables of interest. Additionally, we excluded 291 states as they were represented by too few subjects (< 100). Of these, we further excluded 85 states as no public information on PD could be retrieved for these areas. The final dataset comprised a total of 4,432,440 subjects (*M*_age_ = 43.5 years, *SD* = 12.6, 52.1% females) from 317 states nested within 23 countries (Table 1).

**Table 1:** Sample sizes of participants across the 23 countries included in the analyses

Country	*n*
Australia	208,466
Austria	31,616
Canada	312,082
Chile	108,929
Czech Republic	217,572
Finland	39,376
France	951,095
Germany	189,273
Hungary	108,331
Italy	1,569
Mexico	82,493
Netherlands	55,085
New Zealand	60,340
Norway	12,390
Poland	321,948
Slovak Republic	110,764
South Africa	212,479
Spain	382,147
Sweden	29,401
Switzerland	33,599
Ukraine	407
UK	465,005
USA	498,073
Total	4,432,440

### Measures

#### Population Density

State-level PD data (in persons per square kilometer) were drawn from a variety of sources ranging from reputable databanks such as Knoema (https://knoema.com/) and Eurostat (https://ec.europa.eu/eurostat) to local government statistics boards (e.g., Australian Bureau of Statistics). Data from 2018 were used, and for a handful of states where data were unavailable, we drew from the next closest year (e.g., 2017 or 2019). As the PD data were highly skewed, a logarithmic transformation was performed (Gelfand et al., 2011; Sng et al., 2017).

#### Income

Income was measured using subjects’ self-perceived level of income on a 7-point scale (1 = *very low*; 7 = *very high*) based on local currency.

#### Fertility

Fertility was indexed by the number of children that subjects reported having based on a scale of 0 to more than 3. This scale format minimizes skew and captures the majority of baby-making variance given that the recent global average has been estimated to be 2.5 children per woman (Roser, 2017).

#### Control Variables

Apart from the typical demographic variables of subject age, sex, and education which were available in our dataset, we controlled for several other covariates that have been theorized to be canonical correlates of PD, including state- and country-level per capita GDP as well as country-level economic inequality. These control variables were considered because greater wealth and economic development tend to allow for better healthcare facilities, family planning education, and access to contraceptives, all of which contribute to decisions about having children (Sng et al., 2017). At the same time, fertility is also associated with the availability of healthcare infrastructure and family planning resources given that high levels of adolescent fertility tend to limit the innovation and economic advancement capacities needed for such developments (Luoto, 2019a, 2019b).

As with PD, state-level GDP was obtained through similar sources and included the use of the Organization for Economic Cooperation and Development (OECD) database. Data from 2018 were used, and for states where data were unavailable, we drew from the next closest year. Country-level estimates of GDP were drawn from the World Bank, and data from 2018 were used for all countries.

For national estimates of economic inequality, we used the Standardized World Income Inequality Database (SWIID; https://fsolt.org/swiid/), a comprehensive index that has found widespread use (e.g., Blake et al., 2018; Elgar et al., 2020; Quispe-Torreblanca, et al., 2021) because of its high degree of comparability and extensive coverage across a wide range of countries (Solt, 2020). The database incorporates information from several sources, including the OECD Income Distribution Database, the World Bank’s PovcalNet, and other government statistical boards around the world. Data from 2018 were used for all countries. The SWIID provides a collated Gini value for each country that ranges from 0 to100%, with higher scores indicating a greater degree of economic inequality.

### Analytical Approach

Multilevel mixed-effects models incorporating simple slopes analyses were conducted using the *nlme* package in R, with subjects (Level 1) nested in states (Level 2) nested within countries (Level 3). Parameter estimates were obtained using the maximum likelihood estimator. To test the robustness of the results, we ran an initial model with only the key variables of interest before comparing it to the final models that controlled for theoretically relevant covariates. Demonstrating an unchanging pattern of results across all models would strengthen the validity of the findings and aligns with recent recommendations to guard against false positives (Simmons et al., 2011).

#### Centering of Predictors

Other than income, grand-mean centering was applied to all predictor variables at their respective levels of measurement to facilitate the interpretation of the intercepts. Income was group-mean centered by subtracting its aggregate for each state. As contextual differences can contribute to variances in self-reported measures (e.g., cultural factors influencing how socioeconomic status is perceived; Miyamoto et al., 2018), group-mean centering is a widely accepted method of removing contextual effects arising from state- and country-level differences, leaving only variance that captures individual-level differences in income (see Raudenbush & Bryk, 2002). Similarly, state-level differences in self-reported income were obtained by subtracting the aggregate for each country. All three income variables, one at each level of measurement, were entered into the final model.

#### Multilevel Models

Intraclass correlations were computed to assess the degree of dependence in the data. State- and country-level differences accounted for 0.2% and 1.2% of variance in fertility, respectively. Though seemingly small, even minute levels of dependence can increase the overall type 1 error rate by more than 10–20% when conventional regression techniques are used and sample sizes within clusters are high (Barcikowski, 1981). Hence, hierarchical linear modeling was used to account for this dependence. Intercepts, representing mean fertility, as well as the individual-level income to fertility coefficient, were allowed to vary across states and countries. These constituted the random effects in our final model, which are represented by the equations below.

Individual level:

$$ {\text{Hypothesis}}\;1:\;FERTILITY_{ijk} = b_{0jk} + b_{1jk} \left( {IncomeL1_{ijk} } \right) + X_{ijk} \delta + r_{ijk} $$

$$ {\text{Hypothesis}}\;2:\;FERTILITY_{ijk} = b_{0jk} + b_{1jk} \left( {IncomeL1_{ijk} } \right)\left( {Sex_{ijk} } \right) + X_{ijk} \delta + r_{ijk} $$

State level:

$$ b_{0jk} = \eta_{00k} + S_{jk} \delta + \zeta_{{{\text{0jk}}}} $$

$$ b_{1jk} = \eta_{10k} + \eta_{11k} \left( {PopDensity_{jk} } \right) + \zeta_{1jk} $$

Country level:

$$ \eta_{00k} = \gamma_{000} + C_{k} \delta + u_{00k} $$

$$ \eta_{10k} = \gamma_{100} + u_{10k} $$

$$ \eta_{11k} = \gamma_{110} + u_{11k} $$

At the individual level, *FERTILITY*_ijk_ represents the fertility of subject *i* from state *j* in country *k*. *b*_0*jk*_ represents the average fertility and *b*_1jk_ is the coefficient for the income to fertility relationship, and both were allowed to vary across states. *IncomeL*1_*ijk*_ is the subject’s group-mean-centered income score. Individual-level covariates are represented by the vector term *X*_*ijk*_*δ*, where *δ* is the vector of regression coefficients that accompany a vector of covariate scores, *X*_*ijk*_. Prediction residuals are represented by the random error component, *r*_ijk_.

At the state level, the individual-level intercept, *b*_0jk_, is modeled to be predicted by the average state fertility rate *η*_00*k*_, while a host of state-level covariates are represented by a vector term *S*_*jk*_*δ* comprising the covariate scores and their coefficients. ζ_0*jk*_ is specified as the error component that permits random individual-level intercepts across states. Similarly, *b*_1jk_ is predicted by the fixed effect η_10*k*_, which is the average income coefficient across states, and ζ_1*jk*_, which specifies the random slopes across states. *η*_11*k*_ captures the cross-level interaction in our model by representing the coefficient of state PD that predicts the income to fertility relationship at the individual level. *PopDensity*_*jk*_ is the state score on PD.

At the country level, *η*_00k_ is predicted by *γ*_000_, which represents the average fertility across all countries, alongside the covariate vector term *C*_*k*_*δ* and error term *u*_*00k*_. The state-level income coefficient, *η*_*10k*_, is predicted by the average coefficient across countries, *γ*_100_, and the random error term *u*_10*k*_. Finally, the cross-level interaction coefficient at the state level, *η*_11*k*_, is similarly predicted by the average interaction coefficient across countries, *γ*_110_, and the random error term *u*_11*k*_.

## Results

### Main Effect Models

Consistent with previous findings that densely populated areas lead to slower LHS (e.g., Sng et al., 2017), our data showed that higher state-level PD predicted fewer children (Table 2, Model B), *b* = − 0.010, *SE* = 0.004, *p* = 0.010, thus corroborating the association between crowded environments and lower fertility. Analyses on individual-level income also strongly predicted fertility in the covariates-inclusive model (Table 2, Model B), *b* = 0.034, *SE* = 0.007, *p* < 0.001. However, the effect dropped substantially in the baseline model (Table 2, Model A), suggesting that income’s relationship with fertility was conflated with effects contributed by age, sex, and education, which is unsurprising given their well-documented links with earning power (Bryan & Linke, 1991). Indeed, bivariate correlations revealed associations between income and age (*r* = 0.14, *p* < 0.001), sex (male, *r* = 0.16, *p* < 0.001), and education (*r* = 0.35, *p* < 0.001).

**Table 2:** Results from linear mixed-effects regression models: cross-level interactions (state-level population density × individual-level income)

Variable	Model A: without covariates		Model B: with covariates	
	*b*	SE *b*	*b*	SE *b*
Individual-level variables				
Income L1	0.002	0.007	0.034***	0.007
Age			− 0.005***	0.000
Female			0.268***	0.001
Education			− 0.037***	0.000
State-level variables				
Income L2	− 0.073***	0.010	− 0.067***	0.011
Population density	− 0.008	0.004	− 0.010*	0.004
GDP per capita			0.000	0.000
Country-level variables				
Income L3	− 0.009	0.034	0.044	0.034
Gini index			0.007***	0.001
GDP per capita			− 0.002	0.001
Cross-level interaction				
Population density × Income L1	0.005***	0.001	0.003*	0.001
Intercept	0.017	0.430	0.015	

Values are unstandardized coefficients with standard errors. Income—L1, 2, 3 differentiates individual, state, and country levels of measurement. The positive regression coefficient for “Female” indicates that women reported a higher number of children than men did

^*^p < 0.05, **p < 0.01, ***p < 0.001

These demographic variables were also predictive of fertility. Given that our individual-level variables were tested using a huge sample of subjects (*N* = 4,432,440) as opposed to a smaller sample of states (*N* = 317) or countries (*N* = 23) for the higher-level variables, we interpreted the size of these effects by comparing the standardized coefficients with Cohen’s (1988, 1992) prescribed rules (*β*_small_ = 0.10, *β*_medium_ = 0.24, *β*_large_ = 0.37). In the covariates-inclusive model, age (*β* = − 0.07, *p* < 0.001) and education (*β* = − 0.05, *p* < 0.001) had negative but minute effects on fertility. Sex, however, displayed a relatively larger effect on fertility (*β* = 0.13, *p* < 0.001), indicating that women tended to report having more children than men did. As the data were collected through dating websites, this skew could be the result of either the greater likelihood that women rather than men would gain custody of children during relationship dissolution (Albertini & Garriga, 2011; Stamps, 2002) or that men can get away with not disclosing their existing children since they are less likely to be the primary caretakers (Lamb et al., 1987; Pleck, 1997), resulting in a larger number of women (versus men) with children who were seeking partners. For the higher-level covariates, the Gini index across countries positively predicted number of *b* = 0.007, *SE* = 0.001, *p* < 0.001, indicating that more economically unequal countries had slightly more fertility. GDP per capita was not predictive of fertility at both the state level (*b* = 0.000, *SE* = 0.000, *p* = 0.775) and country level (*b* = -0.002, *SE* = 0.001, *p* = 0.218).

### Moderation by Individual Income

**Fig. 1:**
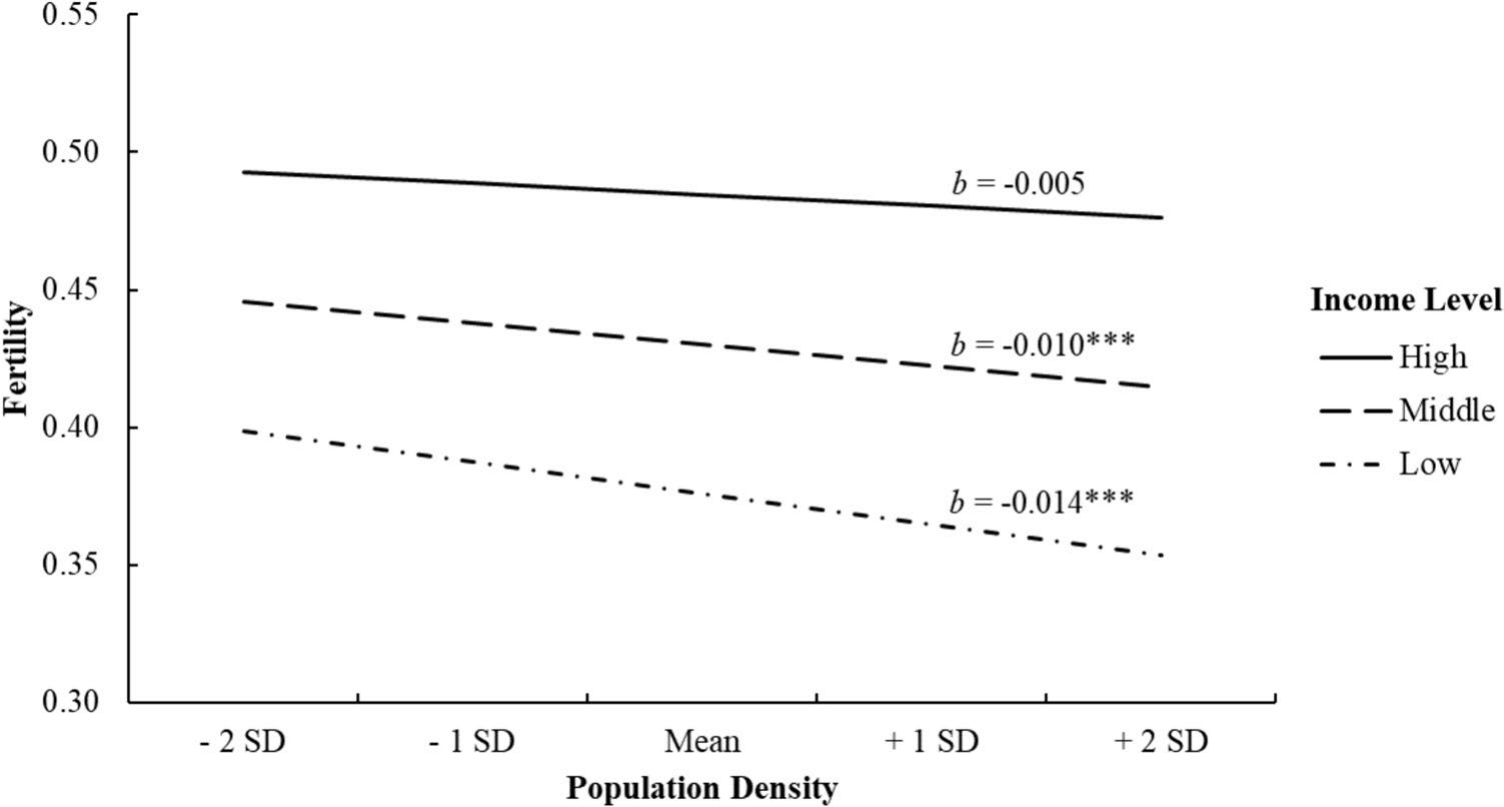
Two-way interaction pattern between population density and income in predicting fertility. Income levels are represented as high (1 SD above the mean), middle (at the mean), or low (1 SD below the mean)

Next, we tested whether individual-level income would moderate the relationship between state-level PD and fertility. As the same pattern of results was observed regardless of whether covariates were included or excluded, we report the full model including covariates. The main effects were qualified by a significant cross-level interaction between individual-level income and state-level PD, *b* = 0.003, *SE* = 0.001, *p* = 0.035 (Table 2, Model B), and simple slopes analyses revealed that higher levels of income were associated with weaker effects of PD on fertility (Table 3). The negative relationship between PD and fertility was significant among low-income individuals (-1 SD from the mean), *b* = − 0.014, *SE* = 0.004, *p* < 0.001, but not among high-income individuals (+ 1 SD from the mean), *b* = − 0.005, *SE* = 0.004, *p* = 0.255. Put differently, the decrease in likelihood to have children from a 1-unit increase in PD was 65% greater for low- relative to high-income individuals. Therefore, as expected, PD more strongly predicted lower reproduction for the economically disadvantaged compared to those who were better off financially (Fig. 1).

**Table 3:** Simple slope effects of the cross-level interaction (state-level population density × individual-level income)

Simple slopes of population density predicting fertility	Model A: without covariates		Model B: with covariates	
	*b*	SE b	*b*	SE b
Low income (− 1 SD)	− 0.016***	0.005	− 0.014***	0.004
Mean income	− 0.008	0.004	− 0.010***	0.004
High income (+ 1 SD)	0.000	0.005	− 0.005	0.004

Values are unstandardized coefficients with standard errors. Simple slopes are analyzed at each level of individual-level income (i.e., income L1)

^*^p < .05, **p < .01, ***p < .001

### Three-way Interaction with Subject Sex

Having found that low-income individuals reproduced less in high-PD areas, we then examined whether this pattern was driven more by men than women by including a three-way, cross-level interaction between income, sex, and PD (Table 4). Analysis on the updated model revealed that the two-way interaction between PD and income remained significant, *b* = 0.005, *SE* = 0.001, *p* < 0.001. More importantly, the higher-order interaction term between the three key variables was indeed significant in predicting fertility, *b* = − 0.003, *SE* = 0.001, *p* < 0.001, suggesting the presence of pattern variations between the lower-order interaction terms depending on the level of a third variable (i.e., a simple interaction).

**Table 4:** Results from linear mixed-effects regression models: cross-level interactions (state-level population density × individual-level income × individual-level sex)

Variable	Model C	
	*b*	SE b
Individual-level variables		
Income L1	0.029***	0.007
Age	− 0.005***	0.000
Female	0.264***	0.001
Education	− 0.036***	0.000
State-level variables		
Income L2	− 0.069***	0.011
Population density	− 0.009*	0.004
GDP per capita	0.000	0.000
Country-level variables		
Income L3	0.044	0.034
Gini index	0.006***	0.002
GDP per capita	− 0.002	0.001
Cross-level interaction		
Population density × Income L1	0.005***	0.001
Population density × Sex	0.016***	0.001
Income L1 × Sex	− 0.043***	0.001
Population density × Income L1 × Sex	− 0.003***	0.001
Intercept	0.424	0.015

Values are unstandardized coefficients with standard errors. Income—L1, 2, 3 differentiates individual, state, and country levels of measurement. The positive regression coefficient for “Female” indicates that women reported a higher number of children than men did

*p < 0.05, **p < 0.01, ***p < 0.001

**Fig. 2:**
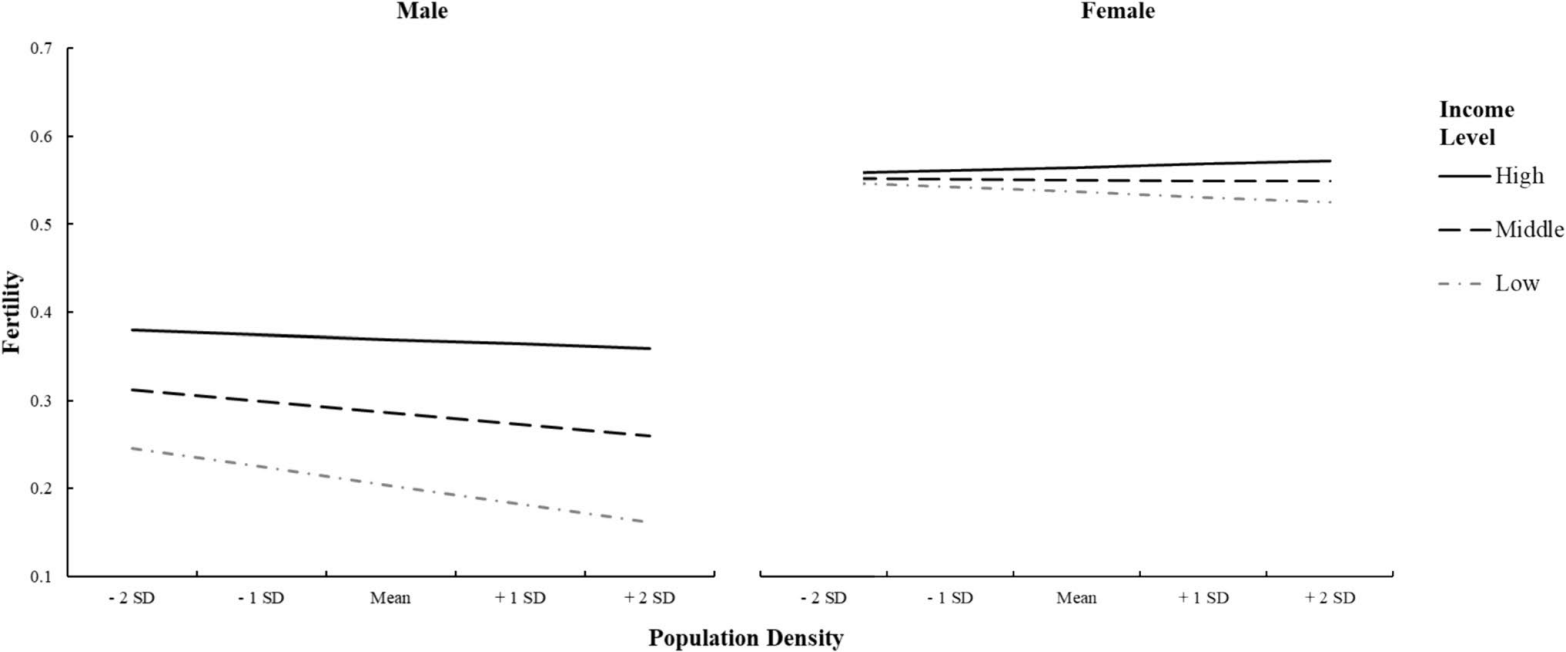
Three-way interaction pattern between population density, income, and sex in predicting fertility. Income levels are represented as high (1 SD above the mean), middle (at the mean), or low (1 SD below the mean). The left and right panels correspond to the simple interaction between population density and income for men and women, respectively

As per predictions, we examined the simple interaction between income and PD at each level of sex (Table 5) and found a stronger simple interaction effect for men, *b* = 0.006, *SE* = 0.001, *p* < 0.001, compared to women, *b* = 0.004, *SE* = 0.001, *p* = 0.007. For each sex, we further dissected this pattern by analyzing the simple slopes between PD and fertility at different levels of income (Table 5). Among high-income individuals (+ 1 SD from the mean), regardless of sex, PD did not significantly predict fertility. However, sex differences were substantially pronounced among low- and middle-income individuals (− 1 SD from the mean and at the mean, respectively), where the inhibitive role of high PD on fertility was stronger for men than for women (Fig. 2). More specifically, the predictive power of PD on the likelihood to have children was 3.7 times higher for men at the bottom of the income ladder, thus supporting our hypothesis that the relationship between low income and high PD on fertility was more pronounced for men.

**Table 5:** Simple interaction and simple slope effects of the three-way cross-level interaction (state-level population density × individual-level income × individual-level sex)

Simple interaction at each level of sex	Model C	
	*b*	SE b
Population density × Income L1		
Male	0.006***	0.001
Female	0.004**	0.001

Simple slopes of population density predicting fertility	Model C	
	*b*	SE b
High income (+ 1 SD)		
Male	− 0.007	0.004
Female	0.005	0.005
Middle income		
Male	− 0.017***	0.004
Female	− 0.001	0.004
Low income (− 1 SD)		
Male	− 0.027***	0.004
Female	− 0.007	0.004

Values are unstandardized coefficients with standard errors. Income L1 represents the individual-level measure

*p < 0.05, **p < 0.01, ***p < 0.001

To account for Galton’s problem of intercorrelations between countries (Pollet et al., 2014; Ross & Homer, 1976). we re-ran all models using one of the more effective methods suggested by Claessens et al. (2023): country-level (Level 3) traits were recalculated as the average score from (a) the focal country and (b) neighboring countries with capital cities within 2,000 km of the capital of the focal country. In the resulting models, the Level 3 predictors of income (Model C: *b* = 0.364, *p* = 0.013) and GDP per capita (Model C: *b* = − 0.006, *p* = 0.010) retained their direction but were now statistically significant. However, no qualitative change to the direction or statistical significance of Level 1 and Level 2 predictors in the model or their interaction terms occurred (see Supplementary Materials for more information), therefore indicating that our findings continue to hold.

## Discussion

The present research contributes to the literature on how LHS and reproductive outcomes respond to individual and environmental factors by examining income as a moderator of the relationship between PD and fertility. Our data revealed that income moderated the negative impact of PD on fertility, thus showing that having adequate resources increased the likelihood of having more children despite the reproductive difficulties imposed by competitive circumstances. Moreover, these patterns being greater for men than for women suggests that financial resources play a larger role in reproductive outcomes for men relative to women. The use of a large sample spread across 317 states and several covariates within a multilevel model provided a powerful test of our hypothesized interactions and ensured the robustness of our results.

### Theoretical Implications

The current study builds on a few important theoretical frameworks. For instance, our findings are consistent with a life history view that reproductive pace depends on factors that determine the payoffs of fast versus slow reproduction (Ellis et al., 2009; Figueredo et al., 2006). Importantly, our multilevel analysis extends prior work that explored the key moderators of the links between ecological features and fertility (e.g., Figueredo et al., 2021; Kuzawa & Bragg, 2012; Luoto et al., 2019; Minkov & Bond, 2015; Rotella et al., 2021) by testing a three-way interaction between individual-level (i.e., income and sex) and state-level factors (i.e., PD). This cross-level interaction approach addresses recently observed ambiguities with the directionality of LHS at different levels of analysis (André & Rousset, 2020; Pollet et al., 2014).

Our findings are also consistent with a social status affordance perspective of mating and reproductive motivation (Yong et al., 2019), which stresses that people’s amenability to marrying and raising a family depends on their preoccupations with social status and resources. More specifically, if people do not have sufficient social status (which determines one's resources) to support the pursuit of reproductive goals, especially in highly competitive societies, they will prioritize competition for social status (e.g., gaining further education, building a career, making money) at the expense of reproductive effort (Li et al., 2011; Yong et al., 2024). Our data indicate that income can be diagnostic of whether there are sufficient affordances to invest in offspring, which has implications for the life history trade-off between competing for resources and having more children.

Demonstrating the greater impact of income on fertility for men relative to women corroborates the vast literature on the importance of social status and resources for male reproductive success (e.g., Buss, 1989; Li, 2007; Yong & Li, 2012; Yong et al., 2022). According to evolutionary theories of mate preferences (Buss & Schmitt, 1993), women’s costly investments in the production of offspring necessitate the selection of mates who can reliably provide protection and resources. Therefore, men with higher status and more resources are more capable of providing for their partners and children and are, thus, likelier to attract mates and raise viable offspring (Fieder & Huber, 2022; Lim, 2021), which underscores the importance of financial resources to men’s mating and reproductive outcomes.

Lastly, the nuances of delaying reproduction and increasing competitive effort as an adaptive trade-off in competitive, densely populated places can also be understood under an evolutionary mismatch framework (Li et al., 2018; Yong et al., 2024). On the one hand, the obsessive pursuit of resources and status appears maladaptive as it lowers fertility at the societal level. On the other hand, because resources and status are crucial to supporting reproductive objectives like mate selection and nurturing competitive offspring, investments in resource and status acquisition are not wasted for the individuals who manage to mate and reproduce. In other words, this trade-off is not necessarily detrimental for those who successfully compete and is therefore still adaptive at the individual level. This resource variability-driven fertility is evident in societies that place a huge premium on wealth and status because the pursuit of education and well-paid, prestigious occupations is essential for men to find a wife and have any children at all (Lim, 2021; Piotrowski et al., 2015; Yong et al., 2024), and women can be similarly affected by the class and educational homogamy in such cultures as people mate assortatively by status (Nitsche et al., 2018; Shafer & Qian, 2010). When having resources and status is a prerequisite to reproduction, the focus on competition for resources and status is ultimately reproductive effort. In modern environments, however, resource and status competition can intensify to such an evolutionarily novel degree that a growing number of people are stuck in competition and experiencing unprecedented levels of competitive stress (see also social status anxiety; Wilkinson & Pickett, 2010) while never progressing to the mating and reproductive phases (Yong et al., 2024). The biggest cities in the world today have evolutionarily novel PDs that are hundreds of thousands of times larger than in ancestral contexts, creating an environment that our evolved mechanisms are not well designed to handle (Li et al., 2018). Because of the inextricable links between resources, status, and reproduction in these competitive modern environments, the pursuit of resources and status—once conducive to mating and reproduction in evolutionarily familiar environments—ironically drives singlehood and childlessness as a rising number of individuals who perceive themselves as chronically lacking in resources and status trade off reproduction for competition permanently (Yong et al., 2024).

### Practical Implications

The present research offers practical ideas on how fertility rates can be more effectively managed. Particularly for countries grappling with below-replacement fertility rates, our findings suggest two approaches that can be applied in tandem: reducing perceptions of PD and increasing perceived resource affordances for reproduction, both of which serve to reduce the perceived need to compete. Insights from the environmental psychology and urban health literatures are instructive for how we may engineer the environment to reduce perceptions of crowdedness. One obvious approach is to incorporate, as an explicit objective, the reduction of the concentrated proximity of persons within areas in urban development projects (Galea et al., 2005), such as by situating neighborhoods and buildings further apart and allowing more space for people based on optimal subnational PDs (Dunbar & Sosis, 2018; Mathur, 2005). Other ways of minimizing cues associated with crowded living include having more natural elements (e.g., parks and other greenery features) and noise reduction features in the built environment, as these have been found to increase people’s perceptions of open space, decreased social presence, and connectedness to nature (Chan et al., 2021, 2023; Evans, 2003; Srinivasan et al., 2003; Takano et al., 2002).

While influencing perceptions of crowdedness by modifying the physical environment is theoretically plausible, this may be very difficult to achieve in places like London or Tokyo where an incredible amount of resources and social engineering would be needed to make such changes without discarding the preexisting infrastructure. A viable alternative is the subtler approach guided by the social status affordance perspective, which suggests that the perceived insufficiency of resources or social status may be mitigated by increasing perceptions of available resources or a wider range of respectable niches in society for people to fill (Rappaport, 2002; Yong et al., 2019). For example, people’s impression of the affordances for starting a family may improve if societies enhance their support systems for raising offspring, such as increasing the availability of affordable childcare and putting in place family-friendly policies (Rovny, 2011). If we also consider that the need to devote time and energy toward competition arises from social status insecurities, then providing more ways for people to achieve social status, such as increasing the prestige of occupations in society (e.g., improving the image or salaries of lower status jobs) or expanding the range of respectable pursuits that people can strive for (e.g., increasing the value placed on activities such as hobbies, volunteering, and pro-environmental behaviors), may lessen people’s preoccupations with social status and shift their attention toward having children.

More broadly, studies have found that PD, competitive stress, and excessive social status striving are negatively associated with happiness and quality of life indicators (Fassio et al., 2012; Gilbert et al., 2009). Therefore, urban planning and cultural transformation initiatives that can reduce perceived crowdedness, increase affordances to pursue a wider range of goals, and lower the urge to compete have significant utility for mental health and wellbeing beyond fertility concerns (Galea et al., 2005).

### Limitations and Further Research

We note several limitations of our research. Despite covering a substantial number of states spanning 23 countries, the sample may not be representative as the countries mostly come from Europe and the Americas (Table 1). This limitation is not trivial given our stated interest in fertility variations across the globe. As some globally comprehensive studies on PD (e.g., Lutz et al., 2006; Rotella et al., 2021; Sng et al., 2017) and wealth (e.g., Borgerhoff Mulder, 1998; Hackman & Hruschka, 2020) suggest that these patterns extend beyond the west to other parts of the world whereas other studies do not (Luoto, 2019a), it is necessary to conduct further research that accounts for a wider range of countries to confirm the generalizability of the relationships we proposed between PD, income, sex, and fertility rates. Similarly, our data were taken from clients of an online dating company, which also presents problems with representativeness given that dating website users are mostly seeking relationships and thus may not have partners or children, while our objective was to assess the number of children that people have ideally within a long-term relationship context. Another issue with this dataset is the sex difference in the number of offspring reported, which could be due to women being more likely than men to have custody of children after a divorce (e.g., Albertini & Garriga, 2011) or men not disclosing their children as they are less likely to be the primary caretakers (e.g., Pleck, 1997), when under more typical circumstances the overall numbers reported by men and women should instead approximate to a more equal amount. Despite these shortcomings, we managed to capture a substantial amount of variance in offspring quantity because of the immense size of the sample, and we were after all most interested in how PD and income would predict fertility differentially for men and women rather than absolute differences in fertility between the sexes. Nevertheless, future research should seek to replicate our findings using samples that are more representative of pair-bonded parents.

While we were able to explain the multilevel interactions underlying reproductive outcomes through a life history lens, our analysis assumed several mechanisms at play but did not test their precise workings. For instance, we proposed that having more income boosts fertility by reducing people’s need to compete for scarce resources, but our model did not include variables such as perceived competition, resource scarcity, or the importance of income for raising a family which would have allowed for more fine-grained analyses of the hypothesized framework. Because of the limited individual-level variables afforded by the online dating dataset, we were unable to explore these mechanisms. We also recognize the shortcomings of using a single-item subjective measure to operationalize having resources, which has several related indicators such as ambition, social status/level, and earning capacity (Buss, 1989; Li et al., 2002) and could arguably be better represented using objective measures (e.g., actual household income). While we were only able to examine self-perceived income level given what was available in the data, we believe the results are defensible given its alignment with theory and wide usage in the literature (e.g., Jonason & Thomas, 2022; Pogosova et al., 2021; Yu, 2019; Zhong et al., 2021), as well as people’s subjective perceptions of what they have often holding value over and above what they actually do have (Yong et al., 2021; also see the relative deprivation hypothesis; Bernstein & Crosby, 1980; Walker & Pettigrew, 1984). Nevertheless, future research should aim to validate the processes by which income and other affordance factors influence the impact of the environment on people’s desire for children by assessing these precise variables with improved instruments. It is also important to note that our analysis was enriched by the inclusion of relevant data (e.g., PD, GDP, Gini) from a wide range of sources, thus making up for these dataset limitations to a considerable extent.

Another possible limitation is that fertility as measured by number of children may not be a reliable proxy of evolutionarily relevant reproductive behavior because contraception—an evolutionarily novel innovation—can decouple sexual activity from reproduction in modern times (Colleran, 2016) and result in different effects of PD on fertility (and on sexual behavior) if contraception was absent. As individual differences in LHS can influence the use of contraception (Miller, 2002), further research on PD and sexual activity may elucidate other pathways by which PD activates or suppresses human psychobehavioral reproductive mechanisms, thus allowing the association between PD and reproduction to be carved more accurately at its evolutionary joints while sidestepping the issue of contraception decoupling sexual behavior and reproduction.

Some degree of phenotypic plasticity was assumed in our hypothesis that people would respond facultatively to PD-induced competition according to the amount of resources they have. This approach implies that people can reflect on their situation in the environment and change their behavior accordingly. However, there may be limits to plasticity as genes and their expression through developmental circumstances play a significant role in shaping LHS variation (e.g., Figueredo et al., 2020; Flatt & Heyland, 2011; Luoto, 2019b). For instance, some polymorphisms in the androgen receptor gene AR, the dopamine receptor gene DRD4, and the 5-HTTLPR VNTR of the serotonin transporter gene have been linked with key features of LHS such as risk appetite and temporal orientation (Minkov & Bond, 2015). From this perspective, individuals who are successful at gaining resources and status may have underlying psychobehavioral tendencies (e.g., industriousness, long-term orientation, conscientiousness, competitiveness) which have been calibrated by genetics and early biosocial experiences to promote resource and status acquisition (Eisenberg et al., 2014; Lukaszewski, 2015; Rimfeld et al., 2016). Even when such individuals have sufficient resources, their psychobehavioral mechanisms would remain active, which might explain why people who are already well off continue wanting to accumulate even more wealth (Carroll, 1998). A related consideration is that the current study does not account for differences in the costliness of offspring, which is important because individuals in impoverished conditions may be inherently fast strategists who would invest less in each child and reproduce regardless of their lack of resources (Pepper & Nettle, 2017). Many developed countries also have society-wide policies such as child allowance and benefits that to some extent allow fast strategists with limited resources to outreproduce slow strategists despite experiencing resource scarcity. Accordingly, the level of resources required for reproduction might vary between individuals at least partly as a function of their LHS. A more complete analysis of LHS in future research should therefore account for genetic components alongside ecological factors (see Yong & Li, 2021, 2022), which would help to elucidate their mutually reinforcing influence on phenotypes.

Finally, we note that our interaction effect sizes are quite small, which may rouse concerns about the practical meaningfulness of our findings and issues with excessive power from using large samples. However, evolutionary theorists have “appreciated for some time that small effects over large populations and periods of time are not bereft of impact” (Jonason & Thomas, 2022, p. 127). For instance, Funder and Ozer (2019) demonstrated that even small correlations have a substantial consequential cumulative effect after a large number of repeated interactions, adding that “in our view, enough experience has already accumulated to make one suspect that small effect sizes from large-*N* studies are the most likely to reflect the true state of nature” (p. 164). Given the small effect sizes that accompany three-way interactions like those in the current investigation, our large-*N* study is warranted as a means to reveal the presence of such effects.

### Conclusion

Through a robust analysis of the state- and individual-level factors underlying LHS and fertility, the current study demonstrated that the association between high PD and low fertility is strongest for people, especially men, with lower income. These findings advance an important multilevel interaction model of LHS that sheds light on the important affordances of financial resources that allow people to focus less on competition for scarce resources and more on having children, particularly for the sex whose reproductive success hinges on the ability to acquire and provide resources.

## Supplementary Information

Below is the link to the electronic supplementary material.

Supplementary file1 (DOC 77 KB)

## Data Availability

A summary and country-level data file and syntaxes for R are provided on the Open Science Framework at https://osf.io/jezm7/. The actual data are proprietary to the Spark Networks Services GmbH but can be shared given some legal considerations. If interested, contact the first author.

## Footnotes

1 It is important to note that this approach is distinct from the classic and more typical conception of human life history strategies, which argues that resource scarcity would drive earlier and increased reproduction because fast strategies are associated with short-term orientation and a “living in the present” mentality instead of accumulating resources for the future at both the population level (e.g., Luoto, 2019a) and the individual level (e.g., de Baca et al., 2016). Nevertheless, there is also some evidence that in an otherwise resource-depleting environment with low GDP and high PD, sudden windfall payments providing economic affluence can set children on a fast life history track (Chang & Lu, 2018). At any rate, these various life history paradigms emphasize different adaptive challenges, with the classic view focusing on faster reproduction as a means to cope with harsh and unpredictable environments whereas the resource-competition view focuses on slower reproduction to cope with competition for scarce resources in high-PD environments. These distinct predictions should be addressed in future research that distinguishes between environmental cues shaping life history strategies and cue-independent adaptations that create individual differences in life history strategies (Figueredo et al., 2004; Galipaud & Kokko, 2020; Minkov & Bond, 2015; Woodley of Menie et al., 2021) and which, according to the classic view, typically predict a negative correlation between resource availability and reproduction (e.g., de Baca et al., 2016; Luoto, 2019a, 2019b).

## Code Availability

The syntaxes are available at https://osf.io/jezm7/.
