# Correction: Income and Sex Moderate the Association Between Population Density and Reproduction: A Multilevel Analysis of Life History Strategies Across 23 Nations

**DOI:** 10.1007/s10508-024-02981-8

**Published:** 2024-08-19

**Authors:** Jose C. Yong, Chun Hui Lim, Peter K. Jonason, Andrew G. Thomas

**Affiliations:** 1 Present Address: Department of Psychology, Northumbria University, Northumberland Building, Northumberland Road, Newcastle Upon Tyne, NE1 8ST UK; 2 Department of Psychology, Stanford University, Stanford, CA USA; 3 Psychology Research Institute, University of Economics and Human Sciences, Warsaw, Poland; 4 School of Psychology, Swansea University, Swansea, UK

**Correction to: Archives of Sexual Behavior** 10.1007/s10508-024-02955-w

Affiliations for two authors were incorrect in the article as originally published and have been corrected to read as follows:

Peter K. Jonason: Psychology Research Institute, University of Economics and Human Sciences, Warsaw, Poland.

Andrew G. Thomas: School of Psychology, Swansea University, Swansea, UK.

In the Population Density, Competition, and Fertility section, the text:

"stimuli, thus supporting future orientation as by which PD slows reproduction and restricts fertility"

 has been corrected to read:

"stimuli, thus supporting future orientation as the mechanism by which PD slows reproduction and restricts fertility"

In the Subjects section, “forover 1 million" has been corrected to read "for over 1 million"

In the Theoretical Implications section, extraneous double quotation marks have been removed from the following portion of text: men with "higher status and more resources

The following entry has been added to the References:

Yong, J. C., & Li, N. P. (2012). Cash in hand, want better looking mate: Significant resource cues raise men’s mating standards. *Personality and Individual Differences, 53*, 55–58.

