# Supplementary material for: Income and Sex Moderate the Association Between Population Density and Reproduction: A Multilevel Analysis of Life History Strategies Across 23 Nations
Source: Arch Sex Behav. 2024 Jul 22;54(1):289–305. doi: 10.1007/s10508-024-02955-w (PMC11782462; doi:10.1007/s10508-024-02955-w)
Supplement: Supplementary file 1 — Supplementary file1 (DOC 77 KB) [file 10508_2024_2955_MOESM1_ESM.doc]

| *Table S1*. Results from Galton-adjusted linear mixed-effects regression models: Cross-level interactions (state-level population density × individual-level income) | | | | | | | |
| --- | --- | --- | --- | --- | --- | --- | --- |
|  |  |  | Model A: Without Covariates | |  | Model B: With Covariates | |
| **Variable** | | | *b* | SE *b* |  | *b* | SE *b* |
|  | Individual-level variables | |  |  |  |  |  |
|  |  | Income L1 | 0.002 | 0.007 |  | 0.034*** | 0.007 |
|  |  | Age |  |  |  | -0.005*** | 0.000 |
|  |  | Female |  |  |  | 0.268*** | 0.001 |
|  |  | Education |  |  |  | -0.037*** | 0.000 |
|  | State-level variables | |  |  |  |  |  |
|  |  | Income L2 | -0.067*** | 0.010 |  | -0.061*** | 0.010 |
|  |  | Population density | -0.008 | 0.004 |  | -0.010** | 0.004 |
|  |  | GDP per capita |  |  |  | 0.000 | 0.000 |
|  | Country-level variables | |  |  |  |  |  |
|  |  | Income L3 | **-0.265**** | **0.071** |  | **0.355*** | **0.136** |
|  |  | Gini index |  |  |  | 0.012*** | 0.003 |
|  |  | GDP per capita |  |  |  | **-0.006*** | **0.002** |
|  | Cross-level interaction | |  |  |  |  |  |
|  |  | Population density × Income L1 | 0.005*** | 0.001 |  | 0.003* | 0.001 |
|  | Intercept | | 0.426 | 0.019 |  | 0.431 | 0.017 |
|  | *Note.* Values are unstandardized coefficients with standard errors. Income - L1,2,3 differentiates individual, state, and country levels of measurement. The positive regression coefficient for “Female” indicates that women reported a higher number of children than men did. **p* < 0.05, ***p* < 0.01, ****p* < 0.001. | | | | | | |

| *Table S2*. Results from Galton-adjusted linear mixed-effects regression models: Cross-level interactions (state-level population density × individual-level income × individual-level sex) | | | | | | |
| --- | --- | --- | --- | --- | --- | --- |
|  |  |  |  | Model C | |  |
| Variable | | |  | *b* | SE b |  |
|  | Individual-level variables | |  |  |  |  |
|  |  | Income L1 |  | 0.030*** | 0.007 |  |
|  |  | Age |  | -0.005*** | 0.000 |  |
|  |  | Female |  | 0.264*** | 0.001 |  |
|  |  | Education |  | -0.036*** | 0.000 |  |
|  | State-level variables | |  |  |  |  |
|  |  | Income L2 |  | -0.063*** | 0.010 |  |
|  |  | Population density |  | -0.010** | 0.004 |  |
|  |  | GDP per capita |  | 0.000 | 0.000 |  |
|  | Country-level variables | |  |  |  |  |
|  |  | Income L3 |  | **0.364*** | **0.132** |  |
|  |  | Gini index |  | 0.011*** | 0.003 |  |
|  |  | GDP per capita |  | **-0.006**** | **0.002** |  |
|  | Cross-level interaction | |  |  |  |  |
|  |  | Population density × Income L1 |  | 0.005*** | 0.001 |  |
|  |  | Population density × Sex |  | 0.016*** | 0.001 |  |
|  |  | Income L1 × Sex |  | -0.043*** | 0.001 |  |
|  |  | Population density × Income L1 × Sex |  | -0.003*** | 0.001 |  |
|  | Intercept | |  | 0.424 | 0.017 |  |
|  |  |  |  |  |  |  |
| *Note*. Values are unstandardized coefficients with standard errors. Income - L1,2,3 differentiates individual, state, and country levels of measurement. The positive regression coefficient for “Female” indicates that women reported a higher number of children than men did.  *p* values: **p* < 0.05, ***p* < 0.01, ****p* < 0.001. | | | | | | |
